# Partial renal deletion of Klotho is not sufficient to impact renal electrolyte handling in distal convoluted tubule specific knock‐out mice

**DOI:** 10.14814/phy2.70297

**Published:** 2025-04-01

**Authors:** Teodora V. Grigore, Quinty M. Leusink, Malou Zuidscherwoude, Caro Bos, Hannes Olauson, Joost Hoenderop

**Affiliations:** ^1^ Department of Medical Biosciences Research Institute for Medical Innovation, Radboud University Medical Center Nijmegen The Netherlands; ^2^ Division of Renal Medicine Department of Clinical Science, Intervention and Technology, Karolinska Institutet Stockholm Sweden

**Keywords:** electrolyte, klotho, mice, NPT2A, TRPV5

## Abstract

Klotho controls renal electrolyte handling by modulating tubular reabsorption of calcium and phosphate through the epithelial calcium channel TRPV5 and sodium phosphate co‐transporter NPT2A. The *Ksp‐KL* ^−/−^ mice have a targeted deletion of *Klotho* in the distal part of the nephron. Considering that the distal convoluted tubule is the most important site for Ca^2+^ regulation in the kidney, *Ksp‐KL* ^−/−^ mice were challenged with a Ca^2+^‐deficient diet for determining the Ca^2+^ handling and pinpointing the *Klotho* levels needed for controlling renal Ca^2+^ handling. The *Ksp‐KL* ^−/−^ mice displayed normal weight and showed unaltered calcium and phosphate levels in serum and 24‐h urine. Expression of calciotropic (*Trpv5*, *Trpv6*) and phosphotropic (*Slc34a1*, *Slc34a2*) genes in the kidneys, duodenum, ileum, and colon were not affected by *Klotho* deletion. In conclusion, our study reports that mice with 18%–93% residual levels of *Klotho* in the kidney exhibit normal electrolyte homeostasis when placed on a low Ca^2+^‐content diet.

## 1 INTRODUCTION

α‐Klotho, further referred to as Klotho, a type I transmembrane protein and co‐receptor for fibroblast growth factor (FGF)‐23, is most abundantly expressed in the kidney and parathyroid gland, tissues that are involved in maintaining calcium (Ca^2+^) homeostasis. The extracellular domain can get cleaved, releasing Klotho into circulation. The principal contributor of circulating Klotho is the kidney (Lindberg et al., 2014), where membrane‐bound Klotho is highly expressed in the renal tubular epithelia, predominantly in the distal convoluted tubules (DCT) and connecting tubule (CT), and to a lower extent in the proximal tubules (PT). Klotho has a broad impact on biological function, as Klotho supplementation or overexpression has been demonstrated to protect against hypertension (Wang & Sun, 2009), endothelial dysfunction (Saito et al., 2000), atherosclerosis, (Yang et al., 2017) and vascular calcification (Hu et al., 2011; Hum et al., 2017).

Klotho is involved in renal electrolyte handling by promoting the reabsorption of Ca^2+^ in the DCT and inhibiting phosphate (Pi) reabsorption in the PT. In the DCT, Klotho anchors the Ca^2+^ transporter transient receptor potential vanilloid‐5 (TRPV5) to the cell membrane surface, preventing its endocytosis (Lee et al., 2021). In the PT, Klotho interacts with the fibroblast growth factor receptor 1c (FGFR1c), forming a FGF23‐specific receptor complex (Agrawal et al., 2021; Kuro, 2019; Urakawa et al., 2006; Yanucil et al., 2022), resulting in reduced Pi serum levels and increased phosphaturia (Gattineni & Baum, 2010; Portale et al., 2015; Takeshita et al., 2018). Together with Klotho, parathyroid hormone (PTH), calcitriol, and FGF23 hormonally regulate the handling of Ca^2+^ and Pi. When serum Ca^2+^ concentration is low, the parathyroid gland secretes PTH, which circulates to the bones and the kidney, where it has direct effects on Ca^2+^ reabsorption. The interaction between PTH and its receptor located in the proximal tubule indirectly elevates calcitriol levels by increasing 1‐alpha‐hydroxylase expression, an enzyme that catalyzes the conversion of 25‐hydroxy vitamin D into 1,25 dihydroxy vitamin D (calcitriol). The increase in calcitriol stimulates intestinal Ca^2+^ absorption (Alexander & Dimke, 2023), regulates TRPV5 expression (Hoenderop et al., 2005) and FGF23 synthesis (Bergwitz & Juppner, 2010). FGF23 and Klotho cause phosphaturia by internalization and degradation of NPT2a (Erben & Andrukhova, 2017), and reduced urinary Ca^2+^ excretion through an increase in TRPV5‐mediated Ca^2+^ reabsorption (Alexander et al., 2009). Canonical FGFR1c‐FGF23‐Klotho signaling is important for Klotho to exercise its effect on renal electrolyte handling; however, Klotho can also act independently. Via FGF23 signaling, Klotho indirectly regulates production of PTH (Hu et al., 2013; Lopez et al., 2011; Mencke et al., 2017), as well as creates a negative feedback loop for modulation of vitamin D production (Forster et al., 2011; Wohrle et al., 2011). *Klotho* and *Fgf23 null* mice highlight the relation between Klotho and FGF23 by displaying similar phenotypes comprising dysfunctional FGF23 signaling, hypercalcemia, hyperphosphatemia, and increased calcitriol levels (Kuro‐o et al., 1997; Larsson et al., 2004; Shimada et al., 2004).

Expression of Klotho decreases gradually during the progression of chronic kidney disease (CKD), while the FGF23 levels increase (Drew et al., 2017; Wolf, 2012). The disturbed Klotho‐FGF23 axis is putatively one of the paramount factors of CKD progression (Hu et al., 2012), as FGF23 levels increase prior to other disturbances in mineral metabolism occurring (Rausch & Foller, 2022). Over the years, Klotho has been the subject of a multitude of studies that investigated its link to pathogenic mechanisms underpinning CKD. It has been demonstrated that Klotho deficiency is consistently associated with heart failure (Cai et al., 2023) and worsens diabetic nephropathy (Lin et al., 2013) and exacerbates vascular calcification (Hu et al., 2011; Lau et al., 2012; Lindberg et al., 2014).

In this study we aimed to assess Ca^2+^ handling in *Ksp‐KL* ^−/−^ mice to increase our understanding of the role of membrane‐bound Klotho and circulating Klotho in these processes. *Ksp‐KL* ^−/−^ mice have previously been shown to have a variable efficiency in Klotho deletion (Olauson et al., 2012), allowing us to explore dose‐dependent effects of Klotho deficiency on renal Ca^2+^ handling. The mice were challenged with a low Ca^2+^ content diet, since the *Ksp‐KL* ^−/−^ mice have a targeted deletion of *Klotho* in the distal nephron including the DCT (Olauson et al., 2012), the major site for regulation of Ca^2+^ excretion. This contrasts with the previous characterization of the *Ksp‐KL* ^−/−^ model, where the mice were placed on normal Ca^2+^ content diets, with normal or high levels of Pi. The mice were placed in metabolic cages to collect blood and 24 h‐urine for biochemical analysis. We investigated serum and urinary levels of electrolytes and the expression of renal and intestinal transporters involved in Ca^2+^ reabsorption, to shed light on the role of Klotho in mineral metabolism and pinpoint the *Klotho* levels needed for controlling renal Ca^2+^ handling.

## 2 RESULTS

### 2.1 Cre‐lox recombination in *Ksp‐KL* ^−/−^ mice has variable efficiency

**FIGURE 1:**
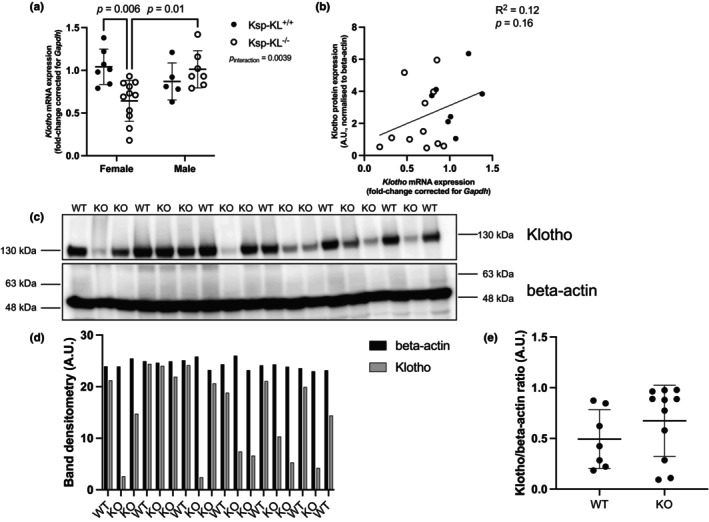
Efficiency of the Cre‐Lox recombination in *Ksp‐KL* ^+/+^ genetic control and *Ksp‐KL* ^−/−^ KO mice fed low Ca^2+^ content chow containing 0.02% Ca^2+^ (w/w). (a) Renal mRNA expression of *Klotho* in *Ksp‐KL* ^+/+^ (represented as filled circles), *Ksp‐KL* ^−/−^ (represented as open circles), where females and males are presented separately; two‐way ANOVA with Tukey's post‐hoc multiple comparison test was conducted. Data shown as mean ± SD. Sample size 30 animals, 7 female WT, 11 female KO, 5 male WT, 7 male KO. (b) Correlation between renal protein levels of Klotho normalized to beta‐actin and renal mRNA expression of *Klotho*; simple linear regression analysis was performed, only females were included in the analysis. Sample size 18 animals, 7 WT, 11 KO. (c) Renal protein levels of Klotho and beta‐actin; only females were included in the analysis. Sample size 18 animals, 7 WT, 11 KO. (d) Densitometry analysis of the Klotho and beta‐actin immunoblots, where beta‐actin levels are represented with black, and Klotho levels are represented with gray. Data shown as bar graph plotting singular data points. Sample size 18 animals, 7 WT, 11 KO. (e) Ratio for densitometry analysis for Klotho/beta‐actin immunoblots. Data shown as mean ± SD. Sample size 18 animals, 7 WT, 11 KO.

There were differences in knock‐out (KO) efficiency between males and females where females had an average of 36% reduction in *Ksp‐KL* ^−/−^ group, while there was no effect in males. The *Klotho* mRNA expression in the *Ksp‐KL* ^−/−^ mice (per total) varied from 18% to 142% using wildtype (WT) mice as reference, whereas females had a *Klotho* mRNA expression ranging from 18% to 93%. Significant differences were noticed in females between the *Ksp‐KL* ^+/+^ group and *Ksp‐KL* ^−/−^ group (*p* = 0.006), as well as between the females and males *Ksp‐KL* ^−/−^ groups (*p* = 0.01) (Figure 1). Since there was no significant Klotho deletion in male *Ksp‐KL* ^−/−^ mice, males were excluded from all further analysis (values from both males and females can be observed in Figure S2). The Klotho protein levels were almost 30% lower in the *Ksp‐KL* ^−/−^ group (2.2 A.U. ±2.0) compared to the *Ksp‐KL* ^+/+^ group (3.3 A.U. ±1.7), when normalized to the loading control beta‐actin. The correlation between renal protein and mRNA Klotho levels (Figure 1b) did not demonstrate the conventional threshold for statistical significance (*p* = 0.16).

### 2.2 *Ksp‐KL* ^−/−^ mice do not show electrolyte disturbances

Mice at 6 weeks of age were placed in metabolic cages to investigate the effect of the 0.02% (w/w) Ca^2+^ challenging diet on *Ksp‐KL* ^−/−^ mice. The low Ca^2+^ content diet did not affect food and water consumption, urine volume, and fecal weight between the control group *Ksp‐KL* ^+/+^ and the *Ksp‐KL* ^−/−^ group (Table 1).

**TABLE 1:** Metabolic cage parameters of *Ksp‐KL* ^+/+^ genetic control and *Ksp‐KL* ^−/−^ KO group fed standard chow with 0.02% (w/w) Ca^2+^ (mean ± SD). Unpaired *t*‐tests were used for comparison between the two groups.

	*Ksp‐KL* ^−/−^ (*n* = 11)	*Ksp‐KL* ^+/+^ (*n* = 7)	*p*
Weight (g)	15.38 (±1.33)	15.03 (±0.85)	0.54
Food intake (g)	2.95 (±0.38)	3.20 (±0.23)	0.14
Water intake (mL)	2.80 (±1.25)	3.43 (±0.29)	0.21
Fecal weight (g)	0.27 (±0.04)	0.27 (±0.03)	0.70
Urine volume (mL)	0.52 (±0.34)	0.58 (±0.32)	0.70

**FIGURE 2:**
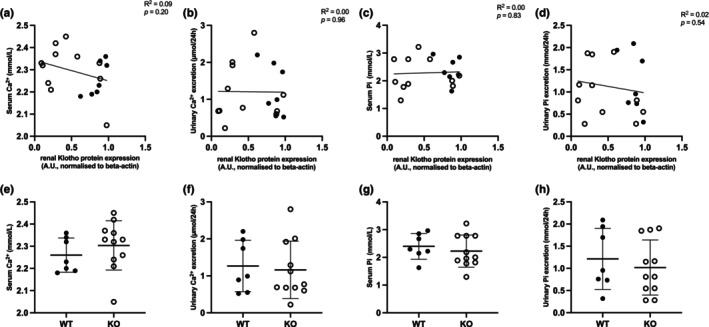
Correlation between the renal Klotho expression and serum Ca^2+^ and Pi concentration and urinary Ca^2+^ and Pi excretion of *Ksp‐KL* ^−/−^ KO (represented as open circles) mice and WT controls (represented as filled circles) fed low Ca^2+^ content chow containing 0.02% Ca^2+^ (w/w); all data are the result of simple linear regression analysis. (a) Serum Ca^2+^ concentration, (b) urinary Ca^2+^ excretion, (c) serum Pi concentration, and (d) urinary Pi excretion. Bar graph comparison between *Ksp‐KL* ^−/−^ KO (represented as open circles) mice and WT controls (represented as filled circles) fed low Ca^2+^ content chow containing 0.02% Ca^2+^ (w/w); all data are the result of student's *t*‐test. (e) Serum Ca^2+^ concentration, (f) urinary Ca^2+^ excretion, (g) serum Pi concentration, and (h) urinary Pi excretion. Only females were included in the analysis. Sample size 18 animals, 7 WT, 11 KO.

To determine whether the low Ca^2+^ content diet and the targeted, partial *Klotho* KO challenged the mice and affected the renal electrolyte handling, Ca^2+^ and Pi levels in serum and urine were measured (Figure 2). There was no statistically significant correlation between renal Klotho protein expression levels and serum Ca^2+^ levels (*R* ^2^ = 0.00, *p* = 0.83) or urinary Ca^2+^ excretion (*R* ^2^ = 0.00, *p* = 0.80). Similarly, Klotho protein expression did not correlate with the Pi levels in serum (*R* ^2^ = 0.00, *p* = 0.83) or in urine (*R* ^2^ = 0.04, *p* = 0.39).

### 2.3 Expression of renal Ca^2+^ and Pi transporters is not correlated with Klotho expression

**FIGURE 3:**
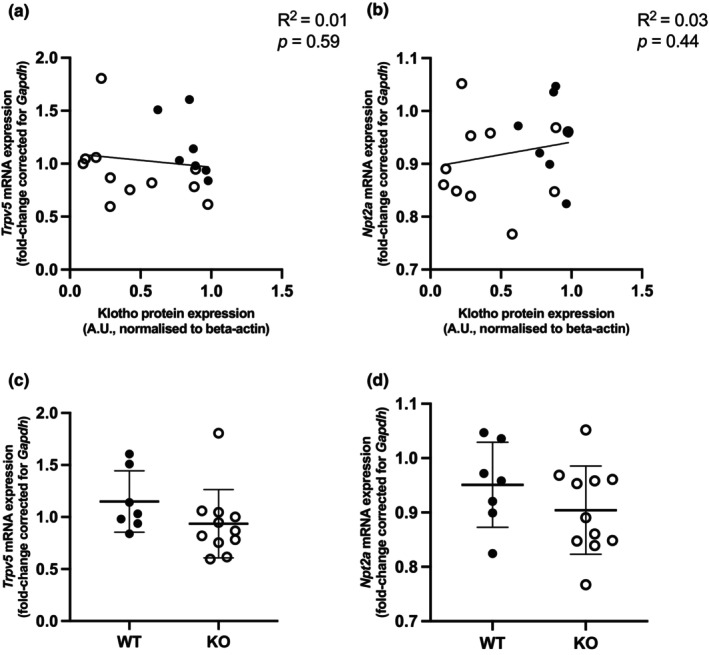
Correlation between the gene expression of Ca^2+^ and Pi‐related genes and *Klotho* in the kidneys of *Ksp‐KL* ^−/−^ (represented as open circles) mice and WT controls (represented as full circles) fed a low Ca^2+^ diet); all data are the result of simple linear regression analysis. Renal mRNA expression of (a) *Trpv5*, (b) *Npt2a*. Bar graph comparison between *Ksp‐KL* ^−/−^ KO (represented as open circles) mice and WT controls (represented as filled circles); data are the result of Mann Whitney test (c) and student's *t*‐test (d). Renal mRNA expression of (c) *Trpv5* and (d) *Npt2a*. Only females were included in analysis. Sample size 18 animals, 7 WT, 11 KO.

Klotho contributes to renal electrolyte homeostasis by regulating the levels of Ca^2+^ and Pi through TRPV5 and NPT2A, respectively. mRNA expression of *Trpv5* and *Npt2a* was not significantly correlated with mRNA Klotho expression (Figure 3), although there is a statistically insignificant trend of decreased *Trpv5* and *Npt2a* expression in the *Ksp‐KL* ^−/−^ mice. Expression of NPT2A did not differ between *Ksp‐KL* ^−/−^ and *Ksp‐KL* ^+/+^ mice (Figure S3).

### 2.4 Expression of intestinal calcio‐ and phosphotropic genes is not correlated with Klotho expression

**FIGURE 4:**
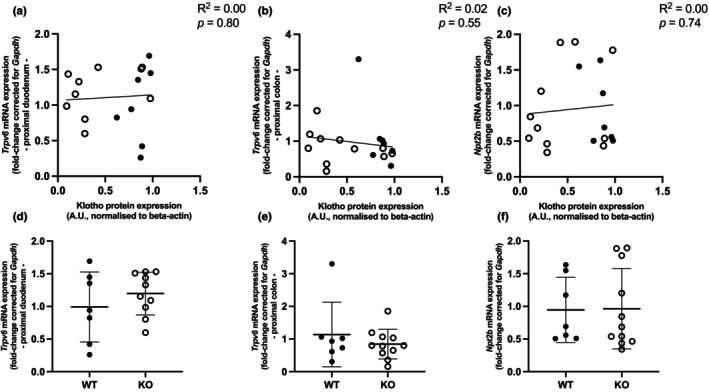
Intestinal gene expression of Ca^2+^ and Pi‐related genes and renal gene expression level of *Klotho* in *Ksp‐KL* ^−/−^ (represented as open circles) mice and WT controls (represented as filled circles) fed low Ca^2+^ diet); all data are the result of simple linear regression analysis. mRNA expression of (a) *Trpv6* in proximal duodenum, (b) *Trpv6* in proximal colon, (c) *Npt2b* in proximal ileum. Bar graph comparison between *Ksp‐KL* ^−/−^ KO (represented as open circles) mice and WT controls (represented as filled circles); data are the result of student's *t*‐test (d, e) and Mann Whitney test (f). Renal mRNA expression of (c) *Trpv6* in proximal duodenum, (d) *Trpv6* in proximal colon and (e) *Npt2b* in proximal ileum. Only females were included in analysis. Sample size 18 animals, 7 WT, 11 KO.

Intestinal absorption is a key player in regulating electrolyte levels. To bring potential compensatory mechanisms to light, mRNA expression levels of genes involved in handling Ca^2+^ and Pi in the intestines were measured. *Trpv6* was measured in the proximal duodenum and proximal colon, while *Npt2b* was analyzed in the proximal ileum. There were no correlations between the expression of *Trpv6* (*R* ^2^ = 0.09, *p* = 0.22) in the duodenum or *Trpv6* (*R* ^2^ = 0.16, *p* = 0.09) in the colon and *Npt2b* (*R* ^2^ = 0.03, *p* = 0.47) with the renal expression of Klotho (Figure 4a–c). The comparison between the control group *Ksp‐KL* ^+/+^ and *Ksp‐KL* ^−/−^ group did not show any significant differences with regard to genes involved in intestinal Ca^2+^ and Pi handling (Figure 4d–f).

## 3 DISCUSSION

In this study, we investigated the influence of Klotho on genes involved in renal Trpv5‐ and Npt2a‐mediated electrolyte handling and in intestinal electrolyte handling. To this end, we used mice with a distal nephron‐targeted *Klotho* KO challenged with a low Ca^2+^ diet. *Ksp‐KL* ^−/−^ mice showed normal electrolyte levels and normal mRNA expression levels of calcio‐ and phosphotropic genes in intestines and kidneys. Based on our results, we conclude that a partial deletion of Klotho in the distal nephron and a low Ca^2+^ diet is not sufficient to affect electrolyte homeostasis.

Global *Klotho* KO mice challenged with a diet low in Ca^2+^, PO_3_^4−^, and vitamin D show hypercalcemia, renal Ca^2+^ wasting, osteopenia, and intestinal Ca^2+^ hyperabsorption (Alexander et al., 2009), highlighting that the Ca^2+^ leak is likely a consequence of Klotho failing to anchor TRPV5 to the membrane. Similarly, a kidney‐specific KO *Klotho* animal model using a different promoter and standard chow was described with increased serum Ca^2+^ levels and pronounced hypercalciuria (Lindberg et al., 2014). We identified some potential reasons for the absence of phenotype in *Ksp‐KL* ^*−/−*^ mice, such as the existence of compensatory mechanisms, which include residual amounts of membrane‐bound Klotho in the DCT that could be sufficient to maintain normocalciuria and normocalcemia, or regulation by circulating Klotho shed from the PT, suggesting either a cross‐talk within the nephron or an integrated synergy between the two expression regions. Although the second putative compensatory mechanism is a previously vehiculated theory (Andrukhova et al., 2014; Chang et al., 2005; Ide et al., 2016), there is not sufficient data to support it so far.

In the previous characterization of the *Ksp‐KL* ^−/−^ mouse model, the animals were placed on a normal diet, containing 0.73% (w/w) Ca^2+^ and 0.52% (w/w) Pi. Interestingly, the *Ksp‐KL* ^−/−^ mice had similar serum Ca^2+^ levels compared to their genetic controls and increased urinary Ca^2+^ excretion. Conversely, the serum PO_3_^4−^ levels were significantly higher in the *Ksp‐KL* ^−/−^ group, with no difference in the urinary PO_3_^4−^ excretion (Olauson et al., 2012). In this study, when placed on a low Ca^2+^ content diet (0.02% w/w), the *Ksp‐KL* ^−/−^ mice did not exhibit significant differences in renal Ca^2+^ and PO_3_^4−^ handling. We speculate that the differences could stem from the Cre‐Lox system, which is commonly associated with limited efficiency (from 30% (Olauson et al., 2012) to >90% (Olauson et al., 2013)) and lack of specificity, as this system is known to exhibit mosaic expression, variable rates of recombination, and sex bias (Choi et al., 2014; Luo et al., 2020; Song & Palmiter, 2018). Notably, the previous characterization shows that the *Trpv5* levels were increased when animals were placed on a normal diet (Olauson et al., 2012). Our findings show no biochemical nor gene expression changes between the *Ksp‐KL* ^−/−^ mice and their genetic controls. While we have no indication that there is compensation at other segments than DCT, we cannot exclude the regulation of channel activity at the protein level or possible post‐translational regulations, for example, intracellular trafficking of channels. Trpv5 is regulated at the protein level, with Klotho anchoring Trpv5 into the cell membrane and thus preventing its endocytosis and renal Ca^2+^ loss (Alexander et al., 2009; Cha et al., 2008; Chang et al., 2005).

Another significant point to be highlighted is the different expression profile of Klotho based on sex. Although it has already been described as a sexually dimorphic protein in the brain (de Mello et al., 2020), with significantly lower levels of Klotho in the cerebrospinal fluid in women than in men (Semba et al., 2014), this characterization was lacking on a renal level. Although there are multiple instances that document increased Klotho levels with males, such as male mice expressing more *Klotho* mRNA (Hsu et al., 2014) or reports that demonstrate a positive association between testosterone and serum Klotho (Dote‐Montero et al., 2019; Glover et al., 2023; Qiao et al., 2023; Zhang et al., 2022), only one recent report describes Klotho as a sexually dimorphic gene in the kidney (Jankowski et al., 2024). These previous reports support our findings that the KO was not as effective in male mice as in females; however, the initial characterization of the Ksp‐KL mouse line (Olauson et al., 2012) described combined data both from female and male mice. Additionally, female mice are often inadequately represented in renal studies due to stronger renoprotective responses (Darvishzadeh Mahani et al., 2021).

In conclusion, our data show that *Ksp‐KL* ^−/−^ mice with 18%–93% expression of *Klotho* in distal tubular segments display normal electrolyte homeostasis when challenged with a low Ca^2+^ content diet. In addition, expression of relevant electrolyte channels was not changed in the kidneys and intestines. Developing better models to further downregulate Klotho would help toward a better characterization of Klotho, from pinpointing the paracrine effects of Klotho on renal electrolyte handling, to contrasting the Klotho‐mediated effects in the DCT versus the PT, as well as establishing the *Klotho* levels needed for controlling the renal Ca^2+^ and Pi handling with a greater degree of accuracy.

## 4 MATERIALS AND METHODS

### 4.1 Generation of kidney specific Klotho knock‐out mice

The generation of the mice with a distal nephron‐specific Klotho deletion using the Cre‐Lox recombination system was previously described by Olauson et al. (2012). Total DNA was extracted from ear biopsies, and PCR amplification was carried out on a T300 Thermocycler (Biometra, Germany) using primers described in Online Resource 1. The PCR products were visualized on a 2% (w/v) agarose gel with SERVA DNA Stain G (39803.02, SERVA, Germany). Presence of Cre recombinase was confirmed through qPCR, by SYBR‐Green (1,708,887, BioRad, California, USA) quantification, using primers described in Online Resource 1, on a CFX96 real‐time PCR detection system (BioRad, California, USA).

### 4.2 Experimental design

The animal experiments described in this paper were performed at the Radboud University Medical Center, Nijmegen, Netherlands. All methods and protocols performed in this study were approved by the Central Animal Laboratory Nijmegen, local Ethics committee of the Radboud University Nijmegen (RU DEC 2020–0006‐004) and the national Ethics committee of the Dutch Central Commission for Animal Experimentation (AVD 10300202010128), and performed according to their guidelines and regulations.

Thirty animals were used: 18 *Ksp‐KL* ^−/−^ mice (11 females, 7 males) and 12 *Ksp‐KL* ^+/+^ mice (7 females, 5 males). *Ksp‐KL* ^+/+^ mice were used as controls and experimental units represent individual mice. Sample size was calculated with a two‐tailed two‐sample *t*‐test power analysis, with a power of 1.0 and alpha of 0.05, based on the outcome measurement of urinary Ca^2+^, using a Java Applet. Mice were weaned at three weeks of age and were immediately placed on synthetic rodent chow (E15000‐047, ssniff Spezialdiaten GmbH, Germany) containing 0.02% (w/w) Ca^2+^ (Table S1), had demineralized water *ad libitum*, and were fed for three weeks until study completion. Animals were housed with maximally 6 mice in standard cages (Eurostandard type IIL) and during the experiments they were individually housed for 24‐h in metabolic cages, after which they were placed back into their own cages. Three weeks after weaning, animals were placed in metabolic cages, where 24‐h urine was collected. Blood sampling was performed by submandibular vein puncture. Three weeks after being placed on the synthetic diet, the animals were placed in metabolic cages, where 24‐h urine was collected. Mice were anaesthetized with 4% (v/v) isoflurane inhalation and exsanguinated by orbital sinus blood collection. Death was confirmed with cervical dislocation. Kidneys were snap‐frozen in liquid nitrogen, and intestines were cleaned with ice‐cold PBS and snap‐frozen in liquid nitrogen. The experimental timeline can be visualised in Figure S1.

### 4.3 Randomization and blinding

The animals included in the experiment were randomly selected by a colleague not involved in the experiment. Similarly, the caging of the animals, their division in metabolic cages, and new labeling were decided through random human selection. The researchers and the handlers were blinded to genotype before the experiment, during the experiment, and during the processing of samples (electrolyte measurements, RNA isolation, cDNA generation and qPCR). The animals were separated by sex to avoid breeding and included littermates. The females were housed randomly, and the males were housed individually or together if they were from the same litter to avoid aggressions. The handling of the cages for feeding, for weight measurement, sample collection, and sacrifice was done randomly.

### 4.4 Electrolyte measurements

Serum and urine Ca^2+^ levels were determined using a chromogen‐based colorimetric assay (P5631‐1G and H6878‐25G, Sigma‐Aldrich, Zwijndrecht, The Netherlands) and read at a wavelength of 570 nm on a BioRad plate reader (BioRad, California, USA). The values obtained were calibrated using Precinorm (Precinorm U, Roche, Switzerland) as a positive control.

Serum Pi levels were determined using a colorimetric assay (MAK307‐1KT, Sigma‐Aldrich, Zwijndrecht, The Netherlands) and read at a wavelength of 620 nm on a BioRad plate reader (BioRad, California, USA). The values obtained were calibrated using Precinorm as a positive control. Urine Pi concentrations were measured at the Faculty of Sciences, Radboud University, using inductively coupled plasma‐mass‐spectrometry (ICP‐MS). Urinary Ca^2+^ and Pi excretion values were determined by correcting for the urine volume collected after the 24‐h placement in metabolic cages.

### 4.5 RNA isolation and cDNA generation

Total RNA was extracted from kidney, proximal ileum, proximal duodenum, and proximal colon using the TRIzol reagent (12,034,977, Invitrogen, Bleiswijk, the Netherlands) according to the manufacturer's protocol. The resulting RNA was treated with 1 U/μg RNA DNase (M6101, Promega, Leiden, the Netherlands) and the concentration and quality were tested with the NanoDrop 2000 (Thermo Fisher Scientific, Breda, the Netherlands). To obtain cDNA, 1.5 μg RNA was reverse transcribed using M‐MLV reverse transcriptase (28,025,013, Invitrogen, Breda, the Netherlands) at 37°C for 1 h.

### 4.6 qPCR

Gene expression levels were quantified as described in Section 2.1, using primer sequences described in Online Resource 1. Gene expression levels were normalized for *Gapdh* expression, and the relative mRNA expression was determined using the Livak method ($2^{-\Delta \Delta C_{T}}$) and illustrated as the fold change of expression compared to the *Ksp‐KL* ^+/+^ group.

### 4.7 Immunoblotting

Frozen kidney tissue was lysed in Triton buffer (50 mmol/L Tris–HCl, 1 mmol/L EDTA, 1 mmol/L EGTA, 150 mmol/L NaCl, 1 mmol/L sodium‐orthovanadate, 10 mmol/L sodium glycerophosphate, 50 mmol/L sodium fluoride, 270 mmol/L sucrose, 10 mmol/L sodium pyrophosphate, 1% (v/v) Triton X‐100, 1% (v/v) PMSF, 0.1% (v/v) leupeptin, 0.1% (v/v) pepstatin A, 0.5% (v/v) aprotinin, complete protease inhibitor cocktail (11,836,170,001, Roche Diagnostics, Switzerland) dissolved in distilled water). Tissues were homogenized on ice and centrifuged at 13000 rpm at 4°C for 15 min. Protein lysate concentration was determined using the bicinchoninic acid (BCA) assay (Thermo Fisher Scientific, The Netherlands). Samples were denatured with Laemmli buffer with 100 mmol/L beta‐mercaptoethanol for 5 min at 95°C, then subjected to SDS‐Page (Criterion TGX, 4–15%, BioRad, California, USA) and transferred to low background polyvinylidene (PVDF) membranes. The membranes were blocked using 5% (w/v) milk, dissolved in Tris‐buffered saline with 0.1% Tween‐20 (TBS‐T) for 1 hour at room temperature. The membranes were immunoblotted overnight at 4°C for Klotho (KM2076, TransGenic Inc., Japan) 1:500 and beta‐actin (A5441, Sigma Aldrich, USA) 1:0000. Subsequently, the blots were washed three times in TBS‐T and incubated with PO‐labeled secondary antibodies (A11006 and A32729, Thermo Fisher Scientific, the Netherlands). After washing three times with TBS‐T, Klotho was visualized using the chemiluminescence SuperSignal West Femto (11,859,290, Thermo Fisher Scientific, the Netherlands), and beta‐actin was visualized using the chemiluminescence SuperSignal West Pico (34,578, ThermoFisher Scientific, the Netherlands) using the ImageQuant LAS 4000 imaging system (GE Healthcare, the Netherlands). Band densities were quantified using the densitometric analysis software ImageJ (version 2.1.0).

### 4.8 Histology and immunohistochemistry

Paraffin‐embedded kidneys were processed into 5 μm‐thick sections and mounted on slides for immunohistochemistry staining. Briefly, slides were deparaffinized for 5 min in xylene in 2 baths, followed by 2 dips in a graded ethanol bath series (100%, 95%, 75%, 50%). Samples were boiled in citrate buffer pH 6.0 for 15 min, permeabilized in 0.1% Tris‐NaCl buffer (0.1 mol/LTris‐HCl pH 7.6, 0.15 mol/L NaCl) and endogenous activity was blocked with 0.3% H_2_O_2_. Blocking was performed in Tris‐NaCl buffer +0.5% Blocking Reagent (Perkin Elmer, USA), then sections were incubated overnight at 4°C with NPT2A primary antibody (Custer et al., 1994) diluted 1:50 in Tris‐NaCl buffer with Blocking Reagent, then washed with Tris‐NaCl buffer with 0.05% Tween‐20, followed by secondary antibody (A21245, Thermo Fisher Scientific, the Netherlands) incubation for 1 h at RT, diluted 1:500. Finally, sections were mounted with Fluoromount‐G (Thermo Fisher Scientific, the Netherlands) and DAPI (Thermo Fisher Scientific, the Netherlands), imaged on Zeiss Axio Observer 7 (Zeiss, Germany) and processed with analysis software Image J (version 2.1.0).

### 4.9 Statistical analysis

All data are presented as arithmetic mean ± SD. Statistical analyses were conducted by two‐way (for plotting data where mice are divided by sex, then two factors appear: genotype and sex) analysis of variance (ANOVA) when comparing more than two groups, with a Tukey's post‐hoc multiple comparison test, by Student's *t*‐test when comparing two normally distributed data sets, and by Mann–Whitney test when comparing two data sets that did not have a Gaussian distribution. Correlations were tested with linear regression analysis. All statistical analysis was done using GraphPad Prism for macOS software, version 10.1.0 (264).

## AUTHOR CONTRIBUTIONS

TG, HO, and JH were involved in the conception and design of the study. TG wrote the work protocols to perform the mouse experiments and was responsible for data management and storage. CB performed the mouse dissections. TG performed the Ca^2+^ and PO_3_^4−^ measurements and real‐time qPCR data. CB and QL performed the immunohistological stainings. TG wrote the manuscript and was responsible for data analysis. TG, MZ, HO, and JH critically revised the manuscript. JH and HO supervised the study. All authors reviewed and approved the final version of the manuscript. JH and HO should be considered joint senior authors.

## FUNDING INFORMATION

This work was supported by grants from the Radboud Institute for Molecular Life Sciences (Radboudumc).

## CONFLICT OF INTEREST STATEMENT

The authors declare no competing interests.

## ANIMAL STUDIES STATEMENT

The authors confirm that the animal experiments performed in this study are reported according to the ARRIVE guidelines.

## Supporting information


Figure S1.

Figure S2.

Figure S3.

Table S1.

## Data Availability

All relevant data are within the manuscript and its supporting information files.
