# Supplementary material for: Partial renal deletion of Klotho is not sufficient to impact renal electrolyte handling in distal convoluted tubule specific knock‐out mice
Source: Physiol Rep. 2025 Apr 1;13(7):e70297. doi: 10.14814/phy2.70297 (PMC11959153; doi:10.14814/phy2.70297)
Supplement: Supplementary file 1 — Figure S1. [file PHY2-13-e70297-s002.pdf]

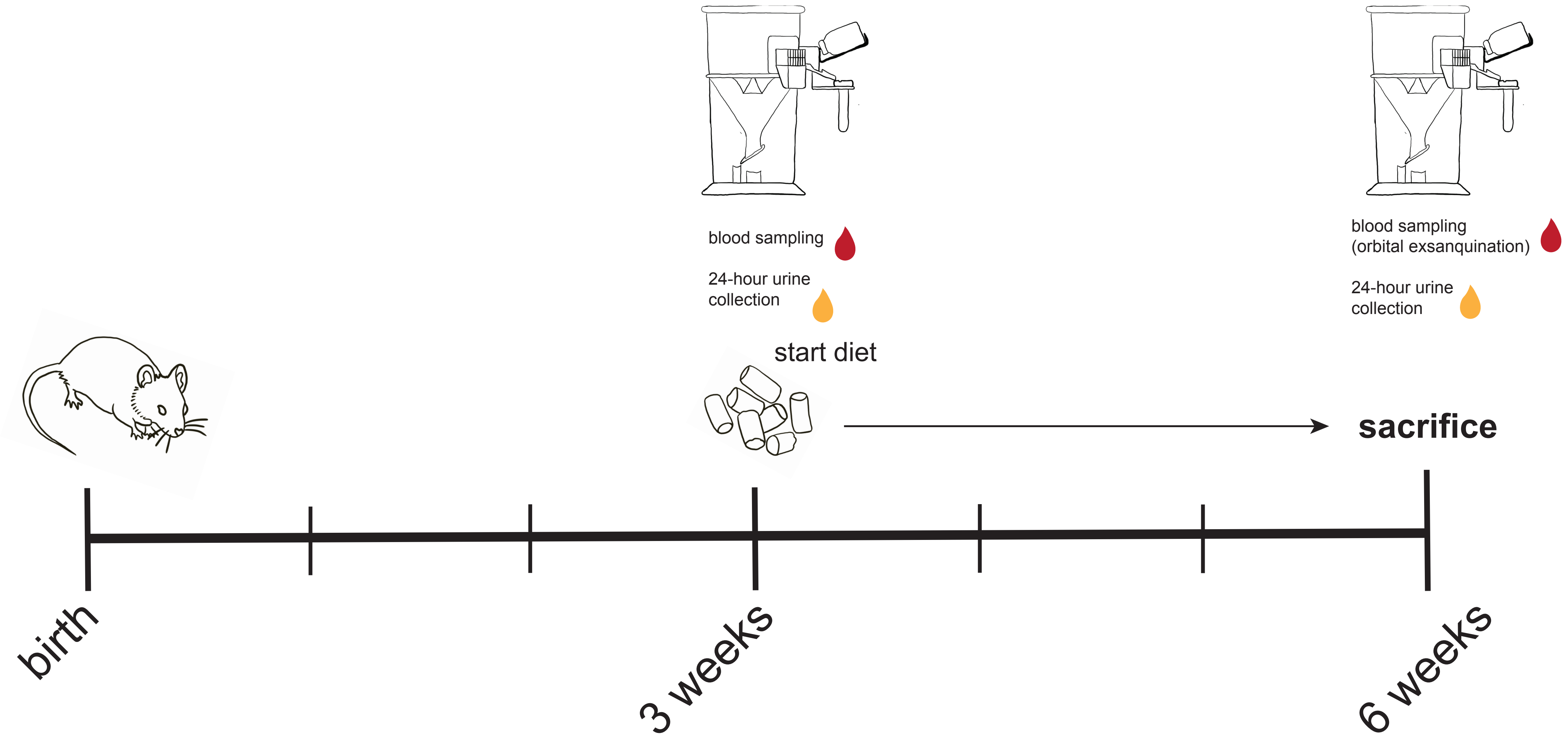

blood sampling
24-hour urine
collection
blood sampling
(orbital exsanquination)
24-hour urine
collection
start diet
sacrifice
birth
3 weeks
6 weeks
