# Supplementary figures and images for: Partial renal deletion of Klotho is not sufficient to impact renal electrolyte handling in distal convoluted tubule specific knock‐out mice
Source: Physiol Rep. 2025 Apr 1;13(7):e70297. doi: 10.14814/phy2.70297 (PMC11959153; doi:10.14814/phy2.70297)

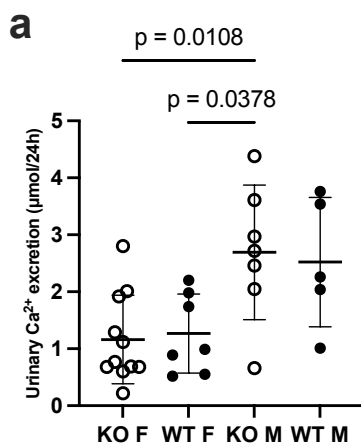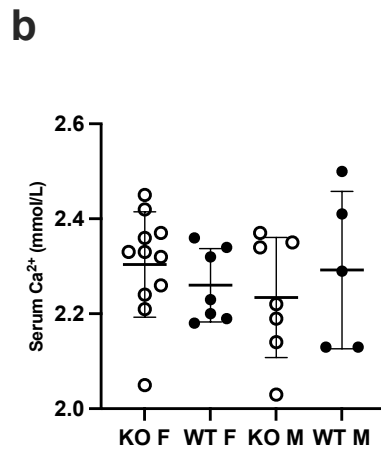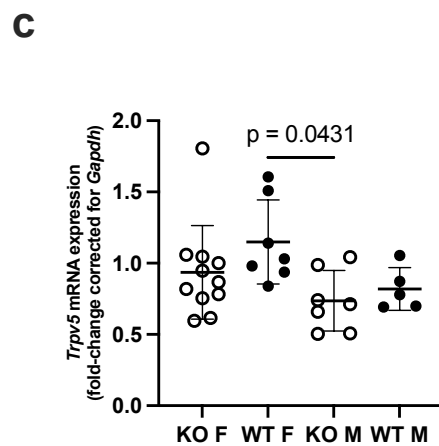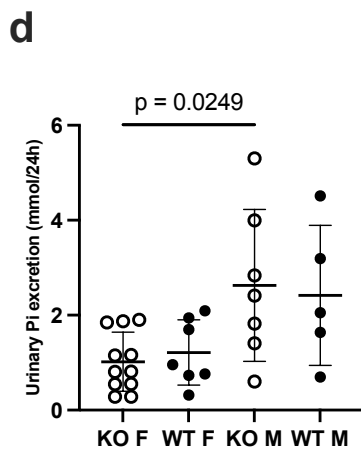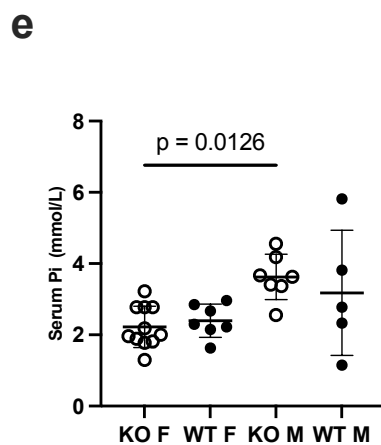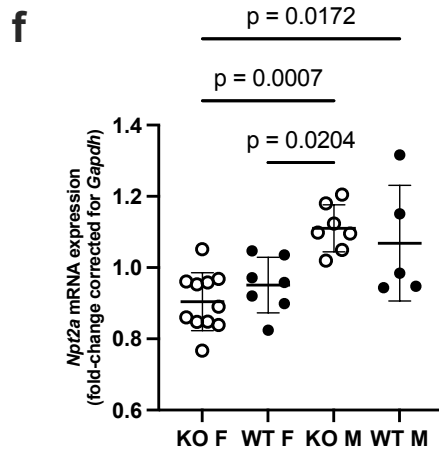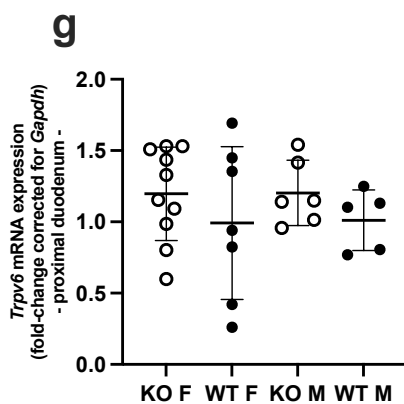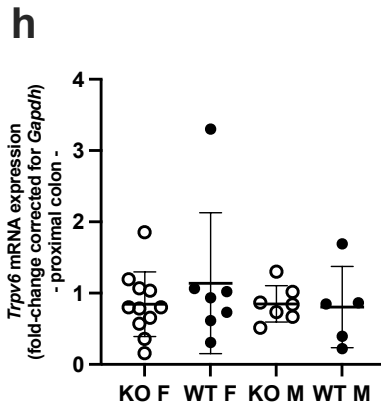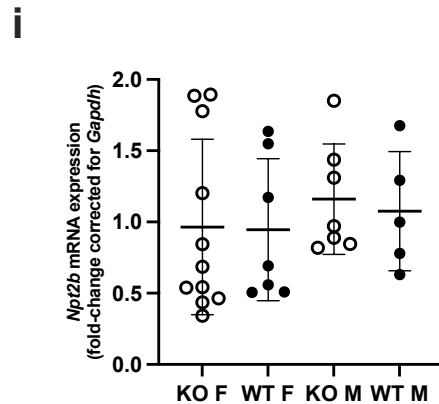

Supplement: Supplementary file 2 — Figure S2. [file PHY2-13-e70297-s003.pdf]

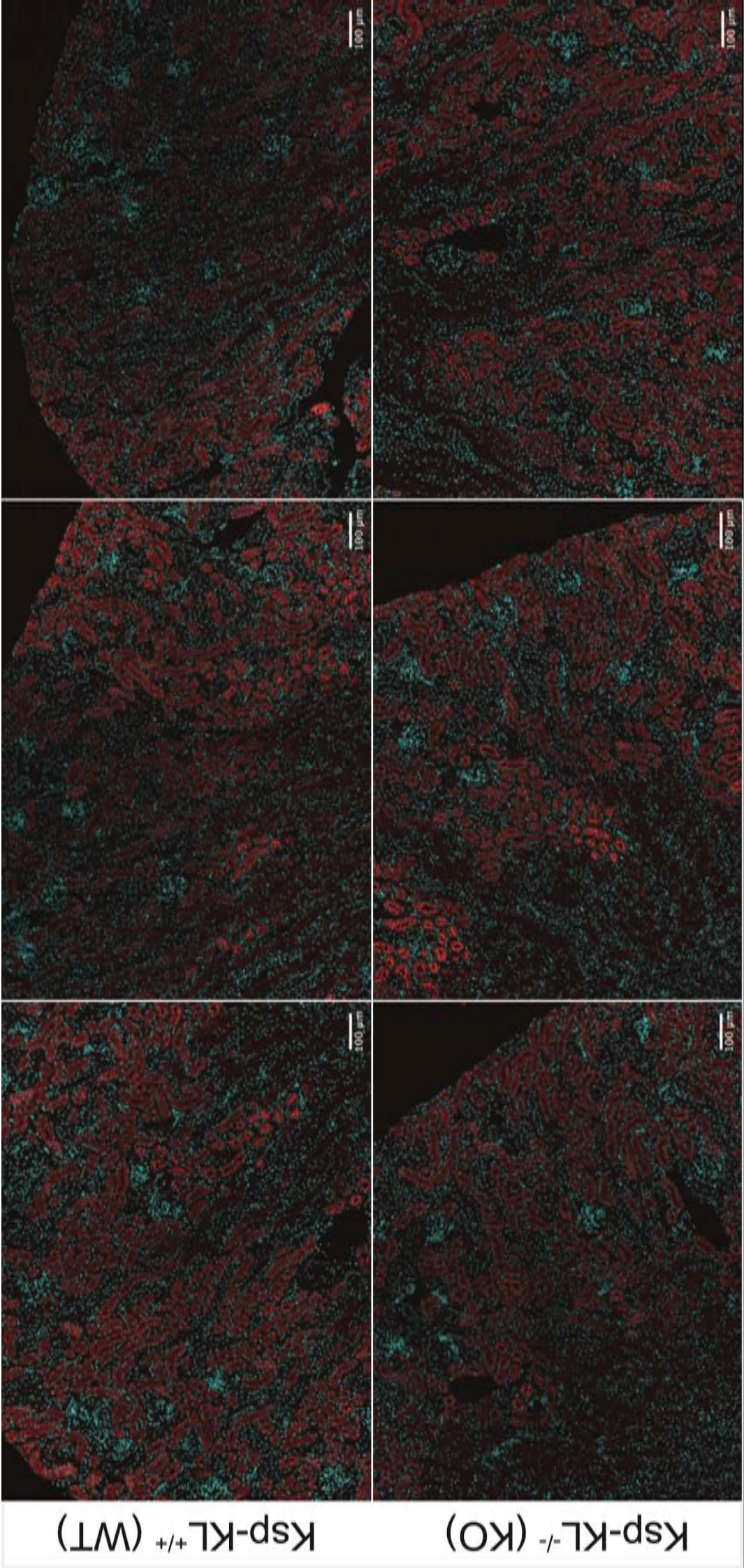

Supplement: Supplementary file 3 — Figure S3. [file PHY2-13-e70297-s001.pdf]
