# Supplementary material for: Partial renal deletion of Klotho is not sufficient to impact renal electrolyte handling in distal convoluted tubule specific knock‐out mice
Source: Physiol Rep. 2025 Apr 1;13(7):e70297. doi: 10.14814/phy2.70297 (PMC11959153; doi:10.14814/phy2.70297)
Supplement: Supplementary file 4 — Table S1. [file PHY2-13-e70297-s004.docx]

Table S1.

| **Minerals** | **%** |
| --- | --- |
| Calcium | 0.02 |
| Phosphorus | 0.65 |
| Sodium | 0.20 |
| Magnesium | 0.23 |
| Potassium | 0.97 |
|  | |
| **Fatty acids** | **%** |
| C 14:0 | 0.02 |
| C 16:0 | 0.50 |
| C 17:0 | 0.01 |
| C 18:0 | 0.15 |
| C 20:0 | 0.02 |
| C 16:1 | 0.01 |
| C 18:1 | 1.03 |
| C 18:2 | 2.11 |
| C 18:3 | 0.23 |
|  | |
| **Amino acids** | **%** |
| Lysine | 1.75 |
| Methionine | 0.75 |
| Cystine | 0.29 |
| Met+Cys | 1.04 |
| Threonine | 0.93 |
| Tryptophan | 0.27 |
| Arginine | 0.83 |
| Histidine | 0.64 |
| Valine | 1.47 |
| Isoleucine | 1.19 |
| Leucine | 2.10 |
| Phenylalanine | 1.10 |
| Phe+Tyr | 2.22 |
| Glycine | 0.42 |
| Glutamic acid | 4.76 |
| Aspartic acid | 1.57 |
| Proline | 2.43 |
| Serine | 1.27 |
| Alanine | 0.64 |
|  | |
| **Vitamins** | **per kg** |
| Vitamin A | 15,000 IU |
| Vitamin D_3_ | 1,500 IU |
| Vitamin E | 150 mg |
| Vitamin K (as MNB) | 20 mg |
| Thiamine (B_1_) | 26 mg |
| Riboflavin (B_2_) | 16 mg |
| Pyridoxine (B_6_) | 16 mg |
| Cobalamin (B_12_) | 30 µg |
| Nicotinic acid | 49 mg |
| Pantothenic acid | 55 mg |
| Folic acid | 16 mg |
| Biotin | 300 µg |
| Choline | 920 mg |
|  | |
| **Trace elements** | **per kg** |
| Iron | 168 mg |
| Manganese | 98 mg |
| Zinc | 67 mg |
| Copper | 14 mg |
| Iodine | 1.2 mg |
| Selenium | 0.2 mg |
